# Predictors of Major Amputation and Mortality in Infected Diabetic Foot Ulcers: A Retrospective Nationwide Inpatient Sample Study

**DOI:** 10.3390/ijerph22091387

**Published:** 2025-09-05

**Authors:** Lior Ben Zvi, David Maman, Michael Margulis, Yaron Berkovich

**Affiliations:** 1 Department of Orthopedics, Carmel Medical Center, Haifa 3436212, Israel; liorbenzvi@gmail.com (L.B.Z.); michailm@012.net.il (M.M.); yaron.berkovich@gmail.com (Y.B.); 2 Rappaport Faculty of Medicine, Israel Institute of Technology, Haifa 2611001, Israel

**Keywords:** diabetic foot ulcers, DFU, cost, length of stay, diabetes

## Abstract

Background: Diabetic foot ulcers (DFUs) affect approximately 15% of diabetic patients and are the leading cause of non-traumatic lower extremity amputations worldwide. This study examines trends in DFU management, predictors of major amputation and in-hospital mortality, and the impact of comorbidities on outcomes. Methods: Using the Nationwide Inpatient Sample (NIS) database (2016–2019), we analyzed non-elective admissions of DFU patients categorized into four treatment groups: no surgery, debridement, minor amputation, and major amputation (below-knee or above-knee). Statistical analyses identified factors associated with major amputation and mortality. Results: A significant increase in minor amputations and debridement was observed between 2016 and 2019, while the number of major amputations declined (*p* < 0.001). Comorbidities varied significantly by treatment type, with dyslipidemia (49.4–51.0%), chronic kidney disease (30.1–44.2%), and hypertension (32.9–47.0%) being the most prevalent (*p* < 0.001). Major amputation was associated with the highest rate of in-hospital mortality (1.00%) and the longest hospital stay (11.2 days) (*p* < 0.001). Logistic regression identified sepsis (OR = 4.9, 95% CI: 4.3–5.6), stroke (OR = 3, 95% CI: 2.1–5.5), and pulmonary embolism (OR = 3.7, 95% CI: 2–6) as key predictors of major amputation, while myocardial infarction (OR = 956, 95% CI: 319–2857) and sepsis (OR = 25, 95% CI: 20–29) were the strongest predictors of mortality (*p* < 0.001). Conclusions: These findings underscore the impact of comorbidities on DFU outcomes and emphasize the need for early intervention to reduce severe complications. Future research should focus on optimizing management strategies for high-risk patients to improve clinical and surgical outcomes.

## 1. Introduction

Diabetic foot ulcers (DFUs) are a severe and increasingly prevalent complication of diabetes mellitus, affecting an estimated 19% to 34% of individuals with diabetes during their lifetime. In the United States alone, approximately 1.6 million new DFU cases are diagnosed each year [1,2,3]. DFUs represent the leading cause of non-traumatic lower-extremity amputations and are associated with significant morbidity, prolonged hospital stays, and elevated healthcare expenditure [2,3,4,5].

The prognosis for patients with DFUs remains poor. Recent studies report a five-year mortality rate ranging from 30% to 56.6%, depending on ulcer severity and associated complications—rates that exceed those observed in many malignancies [3,6,7]. These data highlight the urgent need for timely prevention, risk stratification, and multidisciplinary care strategies.

The progression of DFUs is often exacerbated by systemic comorbidities such as sepsis, hypertension, cardiovascular disease, and chronic kidney disease (CKD) [5,8,9,10]. Among these comorbidities, CKD has been particularly linked to adverse outcomes and increased in-hospital mortality [11,12]. Early identification and proactive management of these underlying conditions are vital to reducing the risk of severe complications, including limb loss and death [9,13,14,15].

Surgical management of DFUs encompasses a spectrum of procedures, ranging from conservative debridement to more invasive interventions such as minor (e.g., toe) and major (e.g., below-knee or above-knee) amputations. Debridement aims to remove devitalized tissue and promote wound healing, whereas amputations are reserved for more advanced cases with extensive infection or tissue necrosis [10,14,15,16,17,18]. Clinical severity and comorbidity burden often dictate the chosen intervention, with more extensive procedures correlating with longer hospital stays, higher healthcare costs, and increased in-hospital mortality [5,8,16].

In recent years, national data have shown a shift in DFU management trends: while the rates of debridement and minor amputations have increased, major amputation rates have declined, particularly between 2009 and 2015 [6]. This change may reflect improvements in early detection and clinical decision-making. Nevertheless, population-level predictors of major amputation and mortality in DFU patients remain inadequately defined.

Major amputation and in-hospital mortality are critical outcome measures in DFU care due to their strong associations with permanent disability, diminished quality of life, and substantial economic burden. Identifying clinical predictors of these outcomes is essential for optimizing risk stratification, guiding treatment decisions, and allocating healthcare resources more effectively.

The aims of this study are as follows: (1) to assess national trends in DFU management between 2016 and 2019; (2) to evaluate the distribution of comorbidities across treatment modalities; and (3) to identify independent predictors of major amputation and in-hospital mortality. We hypothesize that specific clinical variables—including sepsis, CKD, and cardiovascular events—are independently associated with an increased risk of these adverse outcomes.

### Research Questions

What are the trends in DFU management, how do comorbidities influence treatment choices, and what are the key predictors of major amputation and in-hospital mortality?

## 2. Methods

### 2.1. Dataset Acquisition and Inclusion Criteria

This study utilized data extracted from the National Inpatient Sample (NIS) database, the largest publicly available all-payer inpatient care database in the United States. The dataset covered the period from 1 January 2016 to 31 December 2019. The study included all patients who presented to U.S. hospitals with diabetic foot ulcers (DFUs), focusing on non-elective admissions. Patients were categorized into four groups based on the type of treatment they received: no surgery, debridement (soft tissue surgery), minor amputation (various ICD-10 codes for minor amputations), and major amputation (specifically below-knee amputation (BKA) or above-knee amputation (AKA).

### 2.2. Statistical Analyses

Statistical analyses were conducted using SPSS 26 and MATLAB 2024, with a significance threshold set at a *p*-value of less than 0.05. Comorbidities were identified and validated through a comprehensive review of patient-specific ICD-10 codes. Various statistical tests, including logistic regression modeling, were performed to visualize annual trends, discern significant predictors, and derive key statistical insights. Microsoft Excel was employed for data visualization purposes.

Variables were selected for inclusion in multivariate models based on clinical relevance and statistical significance (*p* < 0.05) in univariate analyses. Feature selection was performed using backward stepwise logistic regression to identify independent predictors. Model validation was conducted through internal cross-validation using random subsets of the dataset, and goodness-of-fit was assessed using Hosmer–Lemeshow tests and the area under the receiver operating characteristic curve.

### 2.3. Outcome Measures

The primary outcome measures included the incidence of major amputation and in-hospital mortality among patients with diabetic foot ulcers. Secondary outcomes included the length of hospital stay and total hospital charges. The study also assessed the prevalence of postoperative complications and comorbidities across different treatment groups.

### 2.4. Ethical Considerations

The study was conducted under exemption status granted by the institutional review board, and the requirement for informed consent was waived due to the de-identified nature of the NIS dataset.

## 3. Results

Table 1 provides a detailed overview of the demographic and baseline characteristics of diabetic foot ulcer patients, categorized by the type of procedure they received. The data highlight notable differences in age, gender, payer type, and racial distribution among patients undergoing no surgery, debridement, minor amputation, and major amputation. These differences are statistically significant and underscore the varying profiles of patients based on their treatment options.

### 3.1. Annual Distribution of Procedures for Diabetic Foot Ulcer Patients

**Figure 1:**
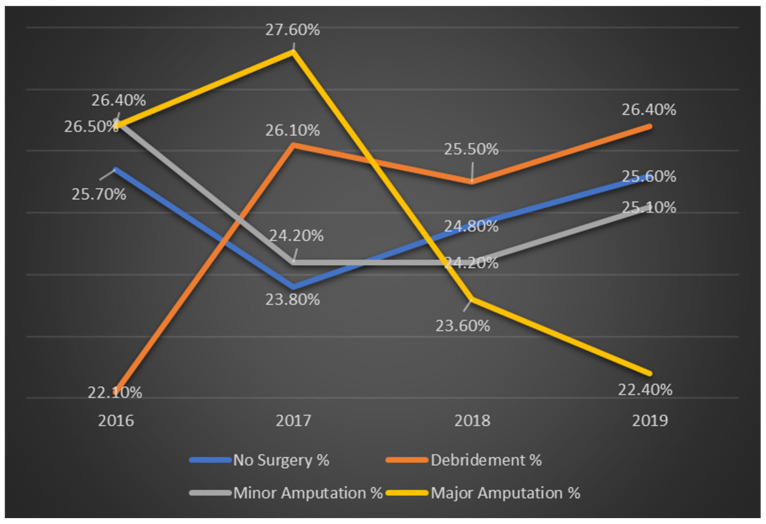
Trends in procedure types for diabetic foot ulcer patients by year in %.

Figure 1 illustrates the annual trends in the types of procedures performed for diabetic foot ulcer patients from 2016 to 2019. The data demonstrate a significant increase in the percentage of patients undergoing debridement, while the percentage of those receiving major amputations has decreased over time. Both trends are statistically significant, with *p*-values of less than 0.001.

### 3.2. Distribution of Comorbidities by Procedure Type

Table 2 displays the prevalence of various comorbidities among diabetic foot ulcer patients based on the type of procedure they underwent. Key observations include that dyslipidemia is most common across all groups, with the highest prevalence among patients undergoing minor amputation. Chronic kidney disease shows a significant increase with the severity of the procedure, peaking in major amputation cases. Hypertension and chronic lung disease are more prevalent in patients who have not undergone surgery. Obstructive sleep apnea and neoplasms are less common but still exhibit significant differences across the treatment groups. Osteoporosis and Parkinson‘s disease are rare and do not show significant statistical differences.

### 3.3. Trends in Postoperative Complications Across Different Treatment Modalities for Diabetic Foot Ulcers

Table 3 shows the incidence of postoperative complications associated with different treatment modalities for diabetic foot ulcers. The data reveal that acute kidney injury is significantly more common in patients undergoing major amputation compared to those receiving no surgery or debridement. Sepsis and stroke are notably rare but exhibit significantly higher rates among major amputation patients. Urinary tract infection and deep vein thrombosis also show statistically significant differences, with higher incidence in patients who underwent more invasive procedures. Pneumonia and ileus are less frequent but still significantly more common in those undergoing major amputation.

### 3.4. Outcomes by Treatment Modality for Diabetic Foot Ulcers

Table 4 details the postoperative outcomes associated with different treatment modalities for diabetic foot ulcers. Mortality rates during hospitalization show a clear increase with the severity of treatment, with major amputations associated with a significantly higher mortality rate of 1.00%, compared to just 0.10% for patients receiving no surgery (*p* < 0.001). The length of hospital stay escalates with more invasive treatments. Patients who underwent major amputations had the longest average stay of 11.2 days, while those with no surgery had the shortest stay of 4.4 days (*p* < 0.001). Total hospital charges follow the same trend, with major amputations resulting in the highest average costs of USD 100,616, compared to USD 34,752 for patients with no surgery (*p* < 0.001).

### 3.5. Multivariate Predictors of Major Amputation

**Figure 2:**
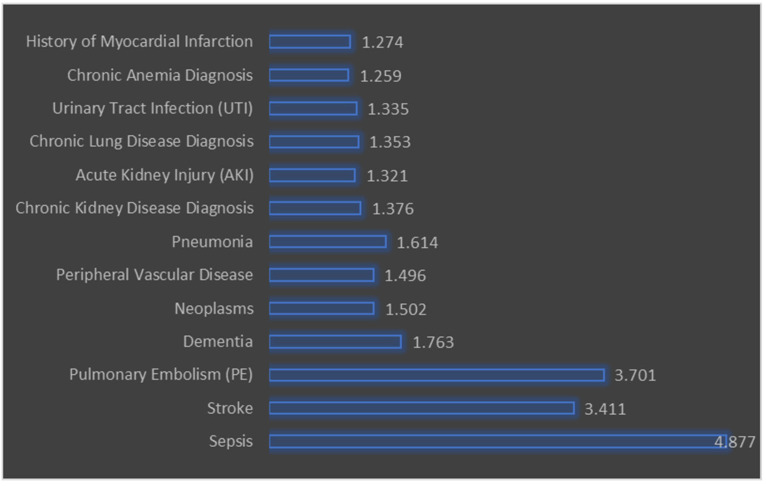
Multivariate predictors of major amputation in diabetic foot ulcers.

Figure 2 illustrates the odds ratios (OR) for various predictors of major amputation in patients with diabetic foot ulcers. The odds ratio quantifies the odds of a particular outcome (major amputation) occurring with the presence of a specific predictor variable compared to the absence of that predictor. The predictors with the highest odds ratios include sepsis (OR = 4.877, 95% CI: 4.256 to 5.588), stroke (OR = 3.411, 95% CI: 2.109 to 5.518), and pulmonary embolism (PE) (OR = 3.701, 95% CI: 2.113 to 6.48). This indicates that these conditions are strongly associated with an increased likelihood of major amputation. Other predictors include dementia (OR = 1.763, 95% CI: 1.511 to 2.056) and neoplasms (OR = 1.502, 95% CI: 1.235 to 1.828), which also show significant associations with major amputation. Peripheral vascular disease (OR = 1.496, 95% CI: 1.365 to 1.64) and pneumonia (OR = 1.614, 95% CI: 1.302 to 2.00) are also significant predictors.

All predictors listed have a *p*-value of less than 0.001, indicating that they are statistically significant predictors of major amputation.

### 3.6. Multivariate Predictors of In-Hospital Mortality

Table 5 presents the OR for various predictors of mortality among patients with DFUs. All predictors listed have a *p*-value of less than 0.001, indicating a high level of statistical significance.

The data show that myocardial infarction (MI) has an exceptionally high odds ratio of 955.978, suggesting an extremely elevated likelihood of death associated with this condition. Similarly, respiratory failure and sepsis also exhibit very high odds ratios of 44.068 and 25.025, respectively, reflecting their substantial impact on mortality. Stroke and pneumonia have somewhat lower but still notable odds ratios of 7.447 and 4.167, indicating their significant roles in predicting death. Additionally, urinary tract infection (UTI) and osteoporosis diagnosis have moderate odds ratios of 3.041 and 2.754, respectively, suggesting that while these conditions are significant predictors, their impact on mortality is less pronounced compared to the conditions with higher odds ratios. Finally, dementia and chronic anemia diagnosis have the lowest odds ratios among the significant predictors, at 1.808 and 1.794, respectively, though they still represent important factors associated with increased mortality.

## 4. Discussion

### 4.1. Main Findings

The primary finding of this study is that while the absolute number of major amputations in DFU management has not significantly changed, the use of minor amputations and debridement has increased over time. This shift may reflect improvements in early detection and localized interventions that help prevent disease progression to more severe forms requiring major limb loss. Similar trends have been reported in prior nationwide analyses, including the study by Armstrong et al., who noted increased use of limb-sparing procedures over the past decade [6,13].

The severity of surgical intervention strongly correlates with poor outcomes. However, an important question remains: are these adverse outcomes driven by the severity of comorbidities, the invasiveness of the intervention itself, or a combination of both? Our data show that patients undergoing major amputations are significantly more likely to experience complications such as urinary tract infections, pneumonia, ileus, and deep vein thrombosis—findings that align with prior studies highlighting the elevated risks of prolonged immobilization and systemic infection in these patients [10,12].

Cardiovascular disease appears to play a central role in the prognosis of DFU patients. Dyslipidemia—an established component of metabolic syndrome—was the most prevalent comorbidity across all treatment groups, emphasizing the need for aggressive systemic disease management [4,6,13,18,19]. CKD, both acute and chronic, was most prevalent among patients requiring major amputations. This finding reinforces the bidirectional relationship between renal dysfunction and poor wound healing in diabetics, as shown in previous studies [20,21,22,23,24].

Of particular note, myocardial infarction (MI) emerged as the strongest independent predictor of in-hospital mortality (OR = 956). Although this figure is statistically accurate based on our model, we acknowledge that such an elevated odds ratio may reflect model instability due to low counts in the non-MI comparison group or collinearity between MI and other high-risk features, such as sepsis or multi-organ failure. Similar findings have been reported in critical care cohorts, where acute MI significantly increases inpatient mortality risk [21]. We have now added a cautionary note regarding this result in both the Abstract and Discussion to clarify its interpretation.

Peripheral arterial disease (PAD) is well established as a major driver of DFU severity, delayed wound healing, and limb loss. While we agree with its clinical importance, the coding of PAD within the NIS database is inconsistent across hospitals and often underreported. For this reason, PAD was excluded from our final regression models.

Our findings are consistent with studies such as those by Zhang et al. and Apelqvist et al., which emphasize the importance of multidisciplinary care and early vascular assessment in reducing amputation rates [22]. The elevated risks associated with sepsis, pulmonary embolism, and stroke—classified in our study as complications rather than comorbidities—highlight the urgency of early medical intervention and aggressive infection control.

Caregivers should anticipate higher mortality rates, longer hospital stays, and increased healthcare costs with more invasive interventions. This information is essential for shared decision-making, helping both patients and clinicians weigh the benefits and risks of available treatment strategies. For instance, Ndip et al. demonstrated that the presence of CKD significantly increases mortality in amputee patients with DFUs, further emphasizing the importance of preoperative optimization [24,25,26,27].

Finally, while this study identifies several independent predictors of poor outcomes, it does not fully elucidate the complex pathways leading to these endpoints. The observed decline in major amputations may not solely reflect improved clinical practice—it could also be influenced by changing hospital admission patterns, coding practices, or demographic shifts. Future research should incorporate longitudinal designs and patient-reported outcome measures to better assess long-term recovery and quality of life.

### 4.2. Strengths and Limitations

This study benefits from the use of a large, nationally representative dataset spanning multiple years, which provides robust insights into DFU trends, surgical outcomes, and hospital-level care patterns across the United States. However, several limitations should be acknowledged. First, the use of administrative data restricts access to detailed clinical information, such as ulcer severity, laboratory values, glycemic control, or outpatient treatment history. Additionally, the NIS does not provide long-term follow-up or patient-reported outcomes, which are essential for assessing the sustained impact of interventions.

Second, while comorbidities and complications were identified using ICD-10 codes, these diagnoses were not validated against medical records [28,29,30,31]. This introduces the potential for misclassification or coding inaccuracies. Third, the cross-sectional design of the dataset precludes any inference of causality between predictors and outcomes [28,29,30,31].

Furthermore, the NIS does not capture important contextual variables such as quality of care, hospital-specific practices, or socioeconomic status. These unmeasured factors may influence treatment decisions and outcomes, leading to residual confounding. For instance, patients from underserved communities or hospitals with limited resources may experience delays in diagnosis or differences in clinical management.

Despite these limitations, our study offers valuable national-level evidence and identifies high-risk profiles that can inform future prospective studies. Future research should aim to validate these findings in clinical cohorts and incorporate longitudinal data to better assess causality and long-term outcomes.

## 5. Conclusions

This nationwide analysis identifies sepsis, stroke, and pulmonary embolism as the strongest independent predictors of major amputation, while myocardial infarction, sepsis, and respiratory failure are the most powerful predictors of in-hospital mortality in DFU patients. These findings reinforce the critical need for early detection and aggressive management of systemic complications in DFU care.

These findings underscore the importance of early intervention and the careful management of comorbidities to mitigate severe outcomes. Further research is warranted to explore strategies for optimizing care and improving long-term outcomes in patients with DFUs.

## Figures and Tables

**Table 1:** Demographic and baseline characteristics of diabetic foot ulcer patients by type of procedure.

Category	No Surgery	Debridement	Minor Amputation	Major Amputation	Sign.
Count	69,315	33,920	58,975	1990	-
Age (Std Dev.)	59.6 (13.0)	57.8 (12.2)	59.1 (12.2)	60.19 (12.2)	*p* < 0.001
Female	33.0%	31.4%	26.4%	22.4%	*p* < 0.001
Primary Expected Payer					
Medicare	48.7%	45.3%	47.0%	57.0%	*p* < 0.001
Medicaid	22.1%	21.3%	19.7%	18.6%	
Private Including HMOs	19.7%	24.0%	24.6%	15.8%	
Self-Pay	6.7%	6.7%	6.1%	4.8%	
No Charge	0.6%	0.4%	0.6%	0.8%	
Other	2.2%	2.4%	2.0%	3.0%	
Race					
White	62.6%	63.2%	62.5%	68.6%	*p* < 0.001
Black	17.2%	16.5%	16.6%	18.3%	
Hispanic	15.3%	14.8%	15.9%	8.7%	
Asian or Pacific Islander	1.0%	1.1%	1.0%	0.3%	
Native American	1.1%	1.7%	1.4%	2.1%	
Other	2.8%	2.7%	2.6%	2.1%	

**Table 2:** Prevalence of comorbidities by procedure type for diabetic foot ulcer patients.

Comorbidity	No Surgery %	Debridement %	Minor Amputation %	Major Amputation %	*p*-Value
Dyslipidemia Diagnosis	49.40%	50.40%	51.00%	49.20%	*p* < 0.001
Hypertension Diagnosis	47.00%	46.80%	43.50%	32.90%	*p* < 0.001
Chronic Kidney Disease Diagnosis	30.10%	32.20%	35.60%	44.20%	*p* < 0.001
Chronic Lung Disease Diagnosis	11.10%	9.30%	9.70%	15.30%	*p* < 0.001
Chronic Anemia Diagnosis	10.20%	11.10%	11.90%	14.30%	*p* < 0.001
Obstructive Sleep Apnea Diagnosis	9.40%	9.50%	9.50%	11.60%	*p* = 0.01
Peripheral Vascular Disease	8.30%	6.20%	9.10%	13.10%	*p* < 0.001
History of Myocardial Infarction	7.50%	7.20%	8.10%	11.80%	*p* < 0.001
Congestive Heart Failure Diagnosis	5.60%	5.10%	4.60%	5.80%	*p* < 0.001
Neoplasms	1.60%	1.70%	1.60%	3.50%	*p* < 0.001
Osteoporosis Diagnosis	1.10%	1.00%	1.10%	1.30%	*p* = 0.81
Parkinson’s Disease Diagnosis	0.70%	0.60%	0.60%	0.50%	*p* = 0.386
Alzheimer’s Disease Diagnosis	0.90%	0.50%	0.50%	0.80%	*p* < 0.001
Dementia	2.50%	1.80%	2.10%	2.80%	*p* < 0.001

**Table 3:** Postoperative complications by procedure type in DFU patients.

Complication	No Surgery %	Debridement %	Minor Amputation %	Major Amputation %	*p*-Value
Acute Kidney Injury	18.50%	20.00%	22.20%	27.90%	*p* < 0.001
Urinary Tract Infection	3.50%	2.50%	2.50%	3.50%	*p* < 0.001
Sepsis	0.60%	1.00%	1.90%	4.30%	*p* < 0.001
Deep Vein Thrombosis	0.70%	1.00%	0.50%	0.80%	*p* < 0.001
Stroke	0.10%	0.10%	0.10%	1.00%	*p* < 0.001
Pneumonia	0.80%	0.70%	1.10%	2.30%	*p* < 0.001
Ileus	0.10%	0.20%	0.10%	0.30%	*p* < 0.001
Respiratory Failure	0.00%	0.10%	0.10%	0.00%	*p* < 0.001
Pulmonary Embolism (PE)	0.10%	0.10%	0.10%	0.30%	*p* = 0.053

**Table 4:** Clinical outcomes of DFU patients by treatment modality.

Measure	No Surgery	Debridement	Minor Amputation	Major Amputation	*p*-Value
Died during hospitalization (%)	0.10%	0.30%	0.30%	1.00%	*p* < 0.001
Length of stay in days (Std Dev.)	4.4 (5.0)	6.2 (5.3)	8.0 (6.2)	11.2 (7.1)	*p* < 0.001
Total charges in USD (Std Dev.)	34,752 (43,954)	54,000 (57,519)	76,719 (76,100)	100,616 (89,982)	*p* < 0.001

**Table 5:** Multivariate predictors of in-hospital mortality in diabetic foot ulcer patients.

Predictor Variable	OR	Lower CI	Upper CI
Myocardial Infarction (MI)	955.978	319.812	2857.203
Respiratory Failure	44.068	23.504	82.678
Sepsis	25.025	20.911	29.949
Stroke	7.447	2.8	19.808
Pneumonia	4.167	2.903	5.984
Urinary Tract Infection (UTI)	3.041	2.276	4.065
Osteoporosis Diagnosis	2.754	1.578	4.81
Dementia	1.808	1.104	2.959
Chronic Anemia Diagnosis	1.794	1.358	2.369
History of Myocardial Infarction	1.561	1.14	2.138

## Data Availability

The original contributions presented in the study are included in the article; further inquiries can be directed to the corresponding author.

## Abbreviations

List of Abbreviations (A–Z):

AKA	Above-Knee Amputation
AKI	Acute Kidney Injury
BKA	Below-Knee Amputation
CKD	Chronic Kidney Disease
DFU	Diabetic Foot Ulcer
DVT	Deep Vein Thrombosis
HMO	Health Maintenance Organization
ICD-10	International Classification of Diseases, 10th Revision
LOS	Length of Stay
MI	Myocardial Infarction
NIS	National Inpatient Sample
OR	Odds Ratio
PAD	Peripheral Arterial Disease
PE	Pulmonary Embolism
UTI	Urinary Tract Infection
